# Changes in Plasma IL-6, Plasma VEGF and Serum YKL-40 During Treatment with Etanercept and Methotrexate or Etanercept Alone in Patients with Active Rheumatoid Arthritis Despite Methotrexate Therapy

**DOI:** 10.4137/bmi.s2300

**Published:** 2009-09-23

**Authors:** Lene Surland Knudsen, Merete Lund Hetland, Julia Sidenius Johansen, Henrik Skjødt, Niels Daugaard Peters, Ada Colic, Karin Grau, Hans Jørgen Nielsen, Mikkel Østergaard

**Affiliations:** 1 Department of Rheumatology, Copenhagen University Hospital at Herlev, Denmark; 2 Department of Rheumatology, Copenhagen University Hospital at Hvidovre, Denmark; 3 Department of Rheumatology, Hjørring County Hospital, Denmark; 4 Department of Rheumatology, Esbjerg County Hospital, Denmark; 5 Department of Rheumatology, Kolding County Hospital, Denmark; 6 Department of Surgical Gastroenterology, Copenhagen University Hospital at Hvidovre, Denmark

**Keywords:** rheumatoid arthritis, etanercept, IL-6, VEGF, YKL-40, biomarkers

## Abstract

Changes in plasma IL-6, plasma VEGF and serum YKL-40 were determined in rheumatoid arthritis (RA) patients during treatment with etanercept alone or in combination with methotrexate. Twenty-five patients with active RA (DAS28 ≥ 3.2) were randomized to receive etanercept (25 mg sc. biweekly) plus methotrexate (n = 12) or etanercept alone (n = 13). Plasma IL-6, plasma VEGF and serum YKL-40 were determined by ELISA. The 3 biomarkers and DAS28 scores were evaluated at baseline and after 4, 8, 12 and 16 weeks of treatment. At inclusion all patients had significantly (p < 0.001) elevated plasma IL-6, plasma VEGF and serum YKL-40 compared to healthy subjects. Eighteen patients responded to treatment (pooled data from both treatment groups), and they had significant (p < 0.05 to p < 0.001) decreases in plasma IL-6, plasma VEGF, serum YKL-40, ESR and DAS28 after 4 weeks of treatment and throughout the study (except serum YKL-40 at week 16). Plasma IL-6 showed the largest reductions. Non-responders had unchanged biomarkers. At week 16 the patients with DAS28 < 3.2 had lower levels compared to baseline values in plasma IL-6 (p = 0.005), plasma VEGF (p = 0.014), and ESR (p = 0.024).

Plasma IL-6, plasma VEGF and serum YKL-40, which reflect different aspects of the inflammatory process, may provide useful information regarding early differentiation of responders from non-responders.

## Introduction

Etanercept, a tumor necrosis factor-alpha (TNF-α) receptor antagonist, in combination with methotrexate has shown to be more effective than etanercept or methotrexate alone for control of disease activity in patients with rheumatoid arthritis (RA).^1^ With the development of new targeted therapies for RA there is a need for more sensitive and specific assessment of treatment response. Molecular markers produced locally in the arthritic joint may reflect the inflammatory and destructive processes in RA, and quantification of these may be a useful adjunct to the clinical evaluation of these patients.

Interleukin-6, produced in the arthritic joint by T and B lymphocytes, macrophages, synoviocytes, osteoblasts and mast- and endothelial cells, plays important roles in the inflammatory processes seen in RA.^2^ The sources of VEGF in RA patients are the synovial lining cells, macrophages, leukocytes, platelets, fibroblasts and endothelial cells.^3^ VEGF stimulates angiogenesis (i.e. the formation of new vessels from pre-existing ones),^4^ which constitute an early event in RA and may lead to cartilage and bone destruction in later stages of the disease.^4^ YKL-40 (chitinase-3-like 1 protein) is produced locally in the arthritic joint by activated macrophages, chondrocytes, synoviocytes and neutrophils.^2^,^5^,^6^ YKL-40 is found to be a possible auto antigen in RA.^7^

We studied the short time changes in plasma IL-6, plasma VEGF and serum YKL-40 in RA patients receiving either etanercept alone or in combination with MTX.

## Materials and Methods

### Patients and therapy

Twenty-five Danish RA patients, who accepted participation in a prospective, randomized, international study (Protocol Number 0881A1-101136, The ADORE study),^8^ were enrolled in this investigator-initiated amendment involving repeated measurements of plasma IL-6, plasma VEGF and serum YKL-40. All patients received methotrexate (for minimum 3 months prior to the study and a stable dose for 6 weeks prior to inclusion), but their disease activity was inadequately controlled (i.e. disease activity score (based on 28 joint counts and the erythrocyte sedimentation rate (ESR), DAS28 ≥ 3.2).^9^ The patients were randomized to receive either etanercept plus methotrexate (n = 12, group A) or etanercept alone (n = 13, group B). Group B patient’s tapered methotrexate during a period of 4 weeks. No patients received intraarticular or systemic steroid injections within 4 weeks before screening. Five patients received a stable dose of oral corticosteroid 4 weeks prior to and throughout the study. Clinical evaluation and blood samples were collected at baseline and after 4, 8, 12 and 16 weeks of treatment.

The study was performed in accordance with the Helsinki Declaration II and approved by the local ethical committee.

### Biochemical analysis

Blood samples were centrifuged within 3 hours after venipuncture and stored at −80 °C until analysis. ESR was determined by routine method. The biomarkers were determined by ELISA: plasma IL-6 and plasma VEGF (R&D Systems, Oxon, U.K), and serum YKL-40 (Quidel, CA, U.S.A.). Samples from each patient were analyzed on the same ELISA plate with internal control samples on each plate to confirm assay precision.

### Healthy subject population

The plasma IL-6 concentration in 318 healthy subjects (median age 48 years, range 18–64, 122 women and 196 men) was median 1.3 ng/l (90th percentile = 3.3 ng/l). The plasma VEGF level in 306 healthy subjects (median age 48 years, range 18–64, 116 women and 190 men) was median 45 ng/l (90th percentile = 109 ng/l). The serum YKL-40 in 245 healthy subjects (median age 49 years, range 18–79, 134 women and 111 men) was median 43 μg/l (90th percentile = 95 μg/l).

### Statistical analysis

Results are given as median, range and percentage changes from baseline. Comparison between and within groups was done by Mann-Whitney and Wilcoxon-Pratt tests, respectively. Correlation was calculated by Spearman’s test. P < 0.05 were considered statistically significant. The statistical tests used are all non-parametric since the interleukin-6, vascular endothelial growth factor and YKL-40 are not normally distributed according to a normal probability plot. Areas under the curve (AUC) values, i.e. weighted means, were calculated for each parameter. The patients were divided according to the EULAR response criteria, into clinical responders (EULAR good or moderate responders) and clinical nonresponders (EULAR non-responders) (www.das-score.nl). For statistical calculations, Sigma Stat (SPSS Inc., Chicago, IL, USA, version 3.1) was used.

## Results

Clinical and biochemical parameters at baseline are given in Table 1. The patients had increased plasma IL-6 (p < 0.001), plasma VEGF (p < 0.008) and serum YKL-40 (p < 0.001) compared to healthy subjects. There were no significant differences in the biomarkers between the two treatment groups at baseline or throughout the study. According to the EULAR response criteria (www.dasscore.nl), 18 patients (group A: n = 10, group B: n = 8) had good (n = 8) or moderate response (n = 10) at the end of the study. Five patients were non-responders (group B) and 2 withdrew due to adverse events (group A). At baseline DAS28 correlated with ESR (rho = 0.52, p = 0.008) and serum YKL-40 (rho = 0.44, p = 0.03), plasma IL-6 with ESR (rho = 0.56, p = 0.003), and HAQ with serum YKL-40 rho = 0.52, p = 0.01), ESR (rho = 0.50, p = 0.01) and DAS28 (rho = 0.72, p < 0.0001).

**Figure 1:**
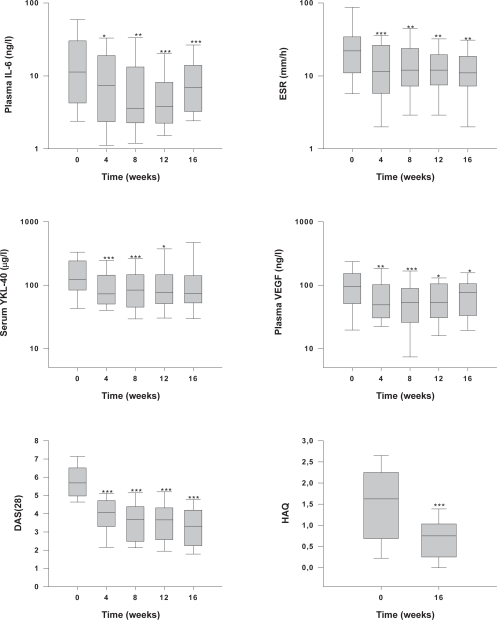
The changes in plasma IL-6, plasma VEGF, serum YKL-40, ESR and DAS28(ESR) in 25 RA patients during the 16-week study period, divided by being EULAR good/moderate responders or EULAR non-responders. Values are given as box and whiskers plots. Boxes indicate 25th, 50th, 75th centiles. Whiskers indicate 5th–95th percentiles. The y-axis is logarithmic for the biomarkers. *, ** and *** indicate statistically significant changes from baseline (Wilcoxon-Pratt): *p < 0.05, **p < 0.01, ***p < 0.001.

Figure 1 illustrates the individual changes in clinical and biochemical markers in the RA patients according to the EULAR DAS28 response criteria. Table 2 shows the percentage decreases compared to baseline values. Significant decreases from baseline values were found in plasma IL-6, plasma VEGF, ESR, and DAS28 at 4, 8, 12 and 16 weeks and in serum YKL-40 at 4, 8 and 12 weeks (p = 0.03 to p < 0.001). Plasma IL-6 and DAS28 showed the most pronounced reductions. All biomarkers, HAQ and DAS28 decreased in both treatment arms, and there was no significantly differences between group A and B. No significant changes were found in the biomarkers of the nonresponders. The only AUC value that correlated was plasma IL6_AUC_ versus ESR_AUC_ (rho = 0.79, p = 0.004).

At week 16, eleven patients had low disease activity (DAS28 < 3.2), and 7 of whose were in remission (DAS28 < 2.6). Patients with DAS28 < 3.2 at week 16 had lower plasma IL-6 levels at this time point compared to baseline level (4.1 vs. 9.6 ng/l, p = 0.005), ESR (8 vs. 16 mm/h, p = 0.024) and plasma VEGF (74 vs. 126 ng/l, p = 0.014). No significant change was found in serum YKL-40. At week 16 plasma IL-6 was significantly higher in RA patients who were in remission compared to healthy subjects (4.1 vs. 1.3 ng/l, p < 0.005) whereas plasma VEGF (81 vs. 45 ng/l, p = 0.318) and serum YKL40 were not (66 vs. 43 μg/l, p = 0.063).

## Discussion

In this study we focused on changes in circulating levels of IL-6, VEGF and YKL-40 in RA patients during anti-TNF-α therapy. TNF-α, IL-1, and IL-17 augment the production of IL-6 by synovial cells and IL-6 is found to be responsible for many of the clinical and biochemical changes in RA.^2^ TNF-α and several pro-inflammatory cytokines (i.e. IL-1, IL-6, IL-8) induce VEGF production and these cytokines are responsible for overexpression of VEGF seen in RA.^3^ IL-1 and TNF-α induces YKL-40 expression and synthesis by chondrocytes and requires activation of nuclear factor kappa B.^10^ In accordance with others^11^–^13^ we found that plasma IL-6, plasma VEGF and serum YKL-40 were significantly increased in RA patients compared to healthy subjects, and that these markers decreased during anti-TNF-α therapy.^5^,^6^,^14^ Etanercept, a construct comprising two human p75 TNF-α receptors coupled to the Fc portion of a monoclonal human antibody (IgG1), binds both TNF-α and TNF-β and thereby reducing cytokine production in patients with RA.^15^ Methotrexate is known to reduce cytokine production through apoptosis of peripherally active T-cells and reduce the number of macrophages in the synovial tissue.^16^ Even though etanercept was able to decrease plasma IL-6, plasma VEGF and serum YKL-40, the level of plasma IL-6 was significantly higher in RA patients in remission than in healthy subject, whereas VEGF and YKL-40 were not. The disease process in the joints not being fully suppressed, despite the low clinical disease activity could explain this. As suggested by others non-responsiveness to TNF-α blockade and residual disease activity, indicate that TNF is not the sole responsible biological target in RA.^17^ Prospective studies including a larger number of RA patients are needed to evaluate if these biomarkers, can provide useful information regarding early differentiation of responders from nonresponders.

## Figures and Tables

**Table 1.** Clinical and biochemical parameters before treatment with etanercept.

**Parameters**	**Median (min–max)**	**Elevated level ¤**
Sex, n (female/male)	17/8	
Age (years)	61 (38–82)	
Disease duration (years)	12.5 (1–48)	
Methotrexate dose (mg, weekly)	15 (12.5–25)	
Swollen joint count (range 0–28)	14 (3–24)	
Tender joint count (range 0–28)	16.5 (2–28)	
HAQ (range 0–3)	1.75 (0–2.875)	
DAS28 (ESR)	5.8 (3.1–7.4)	
Rheumatoid factor (kiu/l, normal level ≤ 17)	72.5 (10–971)	23/25 (92%)
ESR (mm/h, normal level ≤ 20)§	23 (3–96)	13/23 (52%)
Plasma IL-6 (ng/l, normal level ≤ 3.3)	10 (1.8–68)	20/25 (80%)
Plasma VEGF (ng/l, normal level ≤ 109)	92 (7–320)	10/25 (40%)
Serum YKL-40 (μg/l, normal level ≤ 95)	108 (25–777)	17/25 (68%)

§ ESR at baseline is missing in 2 patients.

¤ The proportion of patients with elevated baseline level of the parameter.

**Abbreviations:** HAQ, Health Assessment Questionnaire; DAS28, Disease Activity Score; ESR, Erythrocyte Sedimentation Rate; IL-6, Interleukin-6; VEGF, Vascular Endothelial Growth Factor; YKL-40, Human cartilage glycoprotein 39, CHI3L1.

**Table 2.** Relative decrease in clinical and biochemical parameters compared to baseline values.

	**4 weeks**	**8 weeks**	**12 weeks**	**16 weeks**
**Responders (n = 18)**				
Plasma IL-6	30* (15)	42*** (11)	47*** (13)	28*** (28)
Plasma VEGF	13** (25)	43*** (5)	19* (13)	4* (17)
Serum YKL-40	28*** (7)	33*** (5)	20* (8)	17 (13)
ESR	44*** (4)	35*** (8)	42** (10)	44** (10)
DAS28	33*** (4)	38*** (5)	38*** (5)	43*** (4)
HAQ				48*** (7)
**Non-Responders (n = 5)**				
Plasma IL-6	32^NS^ (18)	−39^NS^ (40)	38^NS^ (3)	−34^NS^ (66)
Plasma VEGF	−65^NS^ (64)	−49^NS^ (14)	−10^NS^ (36)	16^NS^ (44)
Serum YKL-40	20^NS^ (10)	−17^NS^ (9)	−28^NS^ (9)	−16^NS^ (6)
ESR	−10^NS^ (19)	−4^NS^ (37)	−5^NS^ (46)	−33^NS^ (68)
DAS28	20^NS^ (9)	10^NS^ (6)	7^NS^ (13)	14^NS^ (1)
HAQ^a^				100^NS^ (100)

The relative decreases, expressed in%, compared to baseline values are given as mean values (standard error of mean). Negative values indicate an increase compared to baseline level.

* p < 0.05,

** p < 0.01, and

*** p < 0.001.

**Abbreviations:** HAQ, Health Assessment Questionnaire; DAS28, Disease Activity Score; ESR, Erythrocyte Sedimentation Rate; IL-6, Interleukin-6; VEGF, Vascular Endothelial Growth Factor; YKL-40, Human cartilage glycoprotein 39, CHI3L1.

## References

[1] Klareskog L Van der Heijde D de Jager JP Therapeutic effect of the combination of etanercept and methotrexate compared with each treatment alone in patients with rheumatoid arthritis: double-blind randomized controlled trial Lancet 2004 363 675 681 15001324 10.1016/S0140-6736(04)15640-7

[2] Ishihara K Hirano T IL-6 in autoimmune disease and chronic inflammatory proliferative disease Cytokine Growth Factor Rev 2002 13 357 368 12220549 10.1016/s1359-6101(02)00027-8

[3] Ikeda M Hosoda Y Hirose S Expression of vascular endothelial growth factor isoforms and their receptors Flt-1, KDR and neuropilin-1 in synovial tissue of rheumatoid arthritis J Pathol 2001 191 426 433 10918218 10.1002/1096-9896(2000)9999:9999<::AID-PATH649>3.0.CO;2-E

[4] Paleolog EM Angiogenesis in rheumatoid arthritis Arthritis Res 2002 4 Suppl 3 S81 90 12110126 10.1186/ar575 PMC3240151

[5] den Broeder AA Joosten LA Saxne T Long term anti-tumour necrosis factor alpha monotherapy in rheumatoid arthritis: effect on radiological course and prognostic value of markers of cartilage turnover and endothelial activation Ann Rheum Dis 2002 61 4 311 318 11874832 10.1136/ard.61.4.311 PMC1754066

[6] Drynda S Kuhne C Kekow J Soluble tumour necrosis factor receptor treatment does not affect raised transforming growth factor beta levels in rheumatoid arthritis Ann Rheum Dis 2002 61 3 254 256 11830433 10.1136/ard.61.3.254 PMC1754023

[7] Joosten LA Coenen-de Roo CJ Helsen MM Induction of tolerance with intranasal administration of human cartilage gp-39 in DBA/1 mice: amelioration of clinical, histologic, and radiologic signs of type II collagen-induced arthritis Arthritis Rheum 2000 43 3 645 655 10728759 10.1002/1529-0131(200003)43:3<645::AID-ANR22>3.0.CO;2-O

[8] van Riel PL Taggert AJ Sany J Efficacy and safety of combination etanercept and methotrexate versus etanercept alone in patients with rheumatoid arthritis with an inadequate response to methotrexate: the ADORE study Ann Rheum Dis 2006 65 1478 1483 16464988 10.1136/ard.2005.043299 PMC1798368

[9] Prevoo ML van’t Hof MA Kuper HH Modified disease activity scores that include twenty-eight-joint counts. Development and validation in a prospective longitudinal study of patients with rheumatoid arthritis Arthritis Rheum 1995 38 38 44 48 7818570 10.1002/art.1780380107

[10] Recklies AD Ling H White C Inflammatory cytokines induce production of CHI3L1 by articular chondrocytes J Biol Chem 2005 280 50 41213 41221 16234240 10.1074/jbc.M510146200

[11] Swaak AJ van RA Nieuwenhuis E Interleukin-6 (IL-6) in synovial fluid and serum of patients with rheumatic diseases Scand J Rheumatol 1988 17 6 469 474 3266031 10.3109/03009748809098809

[12] Ballara S Taylor PC Reusch P Raised serum vascular endothelial growth factor levels are associated with destructive change in inflammatory arthritis Arthritis Rheum 2001 44 9 2055 2064 11592367 10.1002/1529-0131(200109)44:9<2055::AID-ART355>3.0.CO;2-2

[13] Johansen JS Stoltenberg M Hansen M Serum YKL-40 concentrations in patients with rheumatoid arthritis: relation to disease activity Rheumatol 1999 38 7 618 626 10.1093/rheumatology/38.7.618 10461474

[14] Paleolog EM Young S Stark AC Modulation of angiogenic vascular endothelial growth factor by tumor necrosis factor alpha and interleukin-1 in rheumatoid arthritis Arthritis Rheum 1998 41 7 1258 1265 9663484 10.1002/1529-0131(199807)41:7<1258::AID-ART17>3.0.CO;2-1

[15] Charles P Elliott MJ Davis D Regulation of cytokines, cytokine inhibitors, and acute-phase proteins following anti-TNF-alpha therapy in rheumatoid arthritis J Immunol 1999 163 3 1521 1528 10415055

[16] Mullan R Bresnihan B Disease-modifying anti-rheumatic drug therapy and structural damage in early rheumatoid arthritis Clin Exp Rheumatol 2003 21 Suppl. 31 S158 164 14969069

[17] Smolen JS Maini RN Interleukin-6: a new therapeutic target Arthritis Res Ther 2006 8 Suppl 2 S2 5 10.1186/ar1969 PMC3226077 16899109

